# Supplementary material for: Cardiopulmonary Exercise Testing in Childhood in Late Preterms: Comparison to Early Preterms and Term-Born Controls
Source: J Pers Med. 2022 Sep 20;12(10):1547. doi: 10.3390/jpm12101547 (PMC9605114; doi:10.3390/jpm12101547)
Supplement: Supplementary file 1 [file jpm-12-01547-s001.zip › jpm-1879433-supplementary.pdf]

Table S1: Demographic, anthropometric and lung function data (Late preterms, early preterms with/without BPD, Healthy term-born controls)

	GA= 34-36.6w (late preterm) n=21 1	GA≤30w without BPD n=23 2	GA≤30w with BPD n=15 3	Healthy control born at term n=25 4	p-value *
**Age (years)**	9.94±0.87	9.62±1.35	9.64±0.97	8.84±0.93	$P^3<0.001$ $P^5=0.004$ $P^6=0.031$
**Male (%)**	12 (57%)	11 (48%)	8 (53%)	13 (52%)	P=0.94
**Gestational Age (weeks)**	34.9±1.0	28.8±1.0	27.9±1.3	39.5±1.4	$P^{1,2,3,5,6}<0.001$
**Birth Weight (gr)**	2373±473	1171±248	1003±304	3315±469	$P^{1,2,5,6}<0.001$ $P^3=0.009$
**Oxygen Supplementation (days) median 25-75**	0 [0-1]	14 [3.5-35]	70 [60-84[	0 [0-0]	$P^3=0.30$ $P^4=0.011$ $P^{1,2,5,6}<0.001$
**Ventilation (days) median 25-75**	0[0-0]	5 [2-16]	16 [6-30]	0[0-0]	$P^{1,2,5,6}<0.001$ $P^3=0.41$ $P^4=0.056$
**Height (cm)**	137.4±7.7	135.0±11.2	133.9±7.2	133.3±6.5	P=0.41
**Weight (kg)**	32.6±6.4	34.3±15.7	31.6±7.0	30.9±7.6	P=0.68
**BMI percentiles**	49.5±29.3	52.8±32.3	55.0±34.5	55.5±31.5	P=0.93
**BMI z score [median] 25-75**	0.12 [(-0.84)- (0.73)]	0.05 [(-0.8)- (0.88)]	0.41 [(-0.84)-(1.00)]	0.28 [(-0.55)-(1.09)]	P=0.90
**FEV_1 (L/Sec)**	1.73±0.38	1.67±0.39	1.41±0.30	1.65±0.32	P=0.056
**FEV_1 (% predicted)**	87.5±16.9	87.8±12.5	75.6±14.9	91.0±11.7	$P^2=0.002$ $P^4=0.02$ $P^6=0.021$
**FVC (L)**	2.07±0.40	1.97±0.43	1.72±0.37	1.89±0.37	P=0.07
**FVC (% predicted)**	94.1±13.6	91.9±11.6	84.4±14.3	95.5±12.2	P=0.082

* P^1= 1 vs 2 p^2=1 vs 3 p^3=1 vs 4 p^4=2 vs 3 p^5=2 vs 4 p^6=3 vs 4

GA - Gestational Age; W – weeks; BPD – bronchopulmonary dysplasia; BMI = body mass index; FEV_1 = forced expiratory volume in the first second; FVC = forced vital capacity

Table S2: CPET parameters (for late preterms, early preterms with/without BPD, healthy term-born controls)

	GA= 34-36.6w (late preterm) n=21 1	GA≤30w without BPD n=23 2	GA≤30w with BPD n=15 3	Healthy control born at term n=25 4	p-value*
**Peak $\dot{V}O_2$ Absolute (ml/min)**	1206±248	1084±320	1243±378	1380±260	$P^3=0.02$<br>$P^5=0.005$
**Peak $\dot{V}O_2$ Specific (ml/kg/min)**	37.6±6.8	33.9±10.8	40.2±11.5	45.2±7.4	$P^3=0.038$<br>$P^5<0.001$<br>$P^6=0.031$
**Peak $\dot{V}O_2$ (%Pred)**	90.2±15.1	85.4±20.8	97.3±25.5	112.4±16.9	$P^3<0.001$<br>$P^5<0.001$<br>$P^6=0.016$
**Peak HR (bpm)**	193.4±7	189.5±10.2	190.9±11.5	191.8±9.8	P=0.63
**Peak HR (%pred)**	96.1±3.9	94.2±5.3	94.7±5.7	95.0±5.0	P=0.63
**VE/VCO_2 Slope**	34.4±5.6	36.9±6.9	34.3±5.1	34.4±4.3	P=0.38
**Peak O_2 pulse (%pred)**	94.1±15.4	90.7±21.4	102.8±24.1	118.8±19.1	$P^{3,5}<0.001$<br>$P^6=0.017$
**Peak O_2 pulse (VO_2/HR)**	6.2±1.1	5.7±1.6	6.5±1.7	7.2±1.4	$P^3=0.031$<br>$P^5<0.001$
**Sat. O_2 Pre**	98.7±1.05	99.1±0.90	98.9±0.96	99.1±1.0	P=0.56
**Sat. O_2 Post**	98.5±1.4	98.8±1.02	98.5±1.7	99.1±1.1	P=0.38
**Peak VE (L/min)**	48.8±12.4	44.0±10.6	40.0±13.4	47.5±9.3	P=0.10
**BR (L)**	20.4±11.0	21.2±13.5	17.5±13.3	20.6±12.0	P=0.83
**BR (%)**	28.7±13.5	30.7±17.1	29.3±18.8	29.3±12.9	P=0.98
**Breathing limitation**					
**Low**	9 (43%)	6 (26%)	5 (33%)	3 (12%)	$P^3=0.02$
**Within normal**	12 (57%)	17 (74%)	10 (67%)	21 (88%)	

* P^1= 1 vs 2 p^2=1 vs 3 p^3=1 vs 4 p^4=2 vs 3 p^5=2 vs 4 p^6=3 vs 4

GA - Gestational Age; W – weeks; BPD – bronchopulmonary dysplasia; Peak VO_2 = oxygen uptake at the peak of exercise; HR - heart rate; VE - minute ventilation; VCO_2 - carbon dioxide production; Sat - Saturation; BR – breathing reserve
